# Supplementary material for: High efficiency planar-type perovskite solar cells with negligible hysteresis using EDTA-complexed SnO2
Source: Nat Commun. 2018 Aug 13;9:3239. doi: 10.1038/s41467-018-05760-x (PMC6089874; doi:10.1038/s41467-018-05760-x)
Supplement: Supplementary file 1 — Supplementary Information [file 41467_2018_5760_MOESM1_ESM.pdf]

Supplementary Information

High efficiency planar-type perovskite solar cells with negligible hysteresis using EDTA-complexed SnO_2

Dong Yang[1,2,*], Ruixia Yang[1], Kai Wang[2], Congcong Wu[2], Xuejie Zhu[1], Jiangshan Feng[1], Xiaodong Ren[1], Guojia Fang[4], Shashank Priya[2,*], and Shengzhong (Frank) Liu[1,3,*]

[1]Key Laboratory of Applied Surface and Colloid Chemistry, Ministry of Education; Shaanxi Engineering Lab for Advanced Energy Technology, School of Materials Science and Engineering, Shaanxi Normal University, Xi'an 710119, China. [2]Center for Energy Harvesting Materials and System (CEHMS), Virginia Tech, Blacksburg, VA 24061, United States. [3]Dalian National Laboratory for Clean Energy, iChEM, Dalian Institute of Chemical Physics, Chinese Academy of Sciences, 457 Zhongshan Road, Dalian 116023, China. [4]Key Laboratory of Artificial Micro- and Nano-structures of Ministry of Education of China, School of Physics and Technology, Wuhan University, Wuhan 430072, China.

*e-mail: dongyang@vt.edu; spriya@vt.edu; szliu@dicp.ac.cn

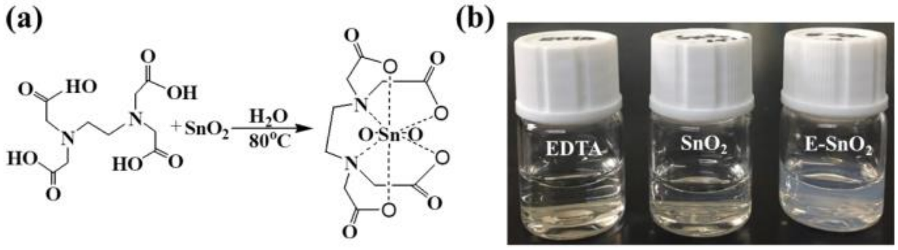

Supplementary Figure 1 | Preparation of the E-SnO_2. (a) Chemical reaction of EDTA and SnO_2 in aqueous solvents at 80 °C. **(b)** Photographs of EDTA, SnO_2 and E-SnO_2 samples.

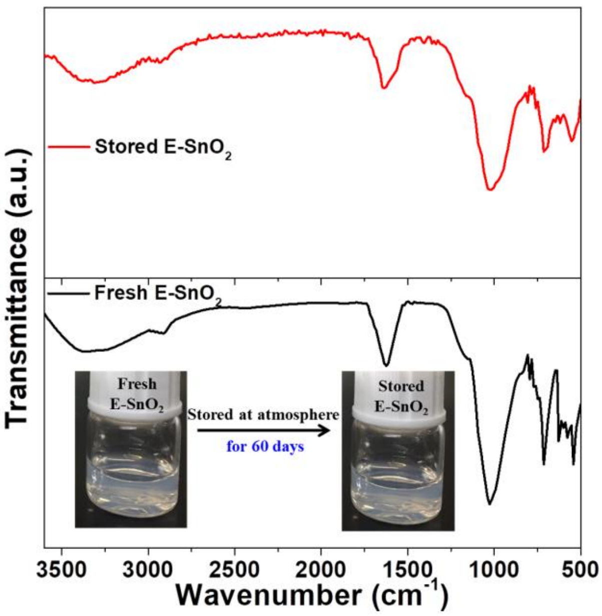

Supplementary Figure 2 | The stability of E-SnO_2. The FTIR and photographs of the E-SnO_2 solution taken under fresh condition and after it was stored in ambient for 60 days.

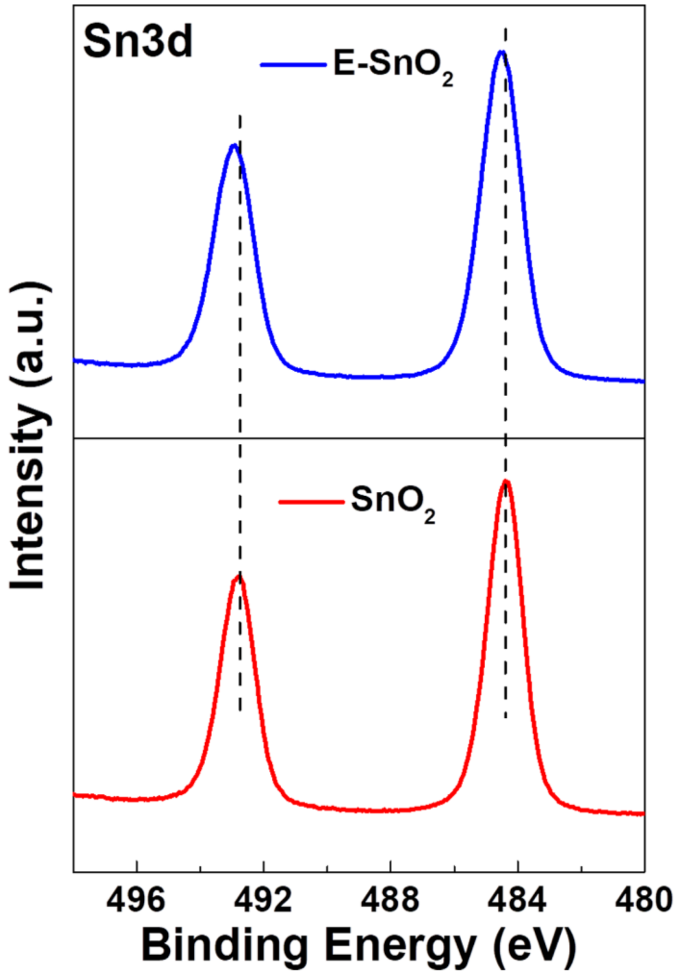

Supplementary Figure 3 | XPS spectra of Sn $3d_{5/2}$ of SnO_2 and E-SnO_2 films. It is obvious that the Sn 3d peaks of E-SnO_2 shift to high binding energy by ~0.16 eV in contrast to SnO_2, which means that Sn atoms are bonded with O and N atoms in EDTA to form chelate products.

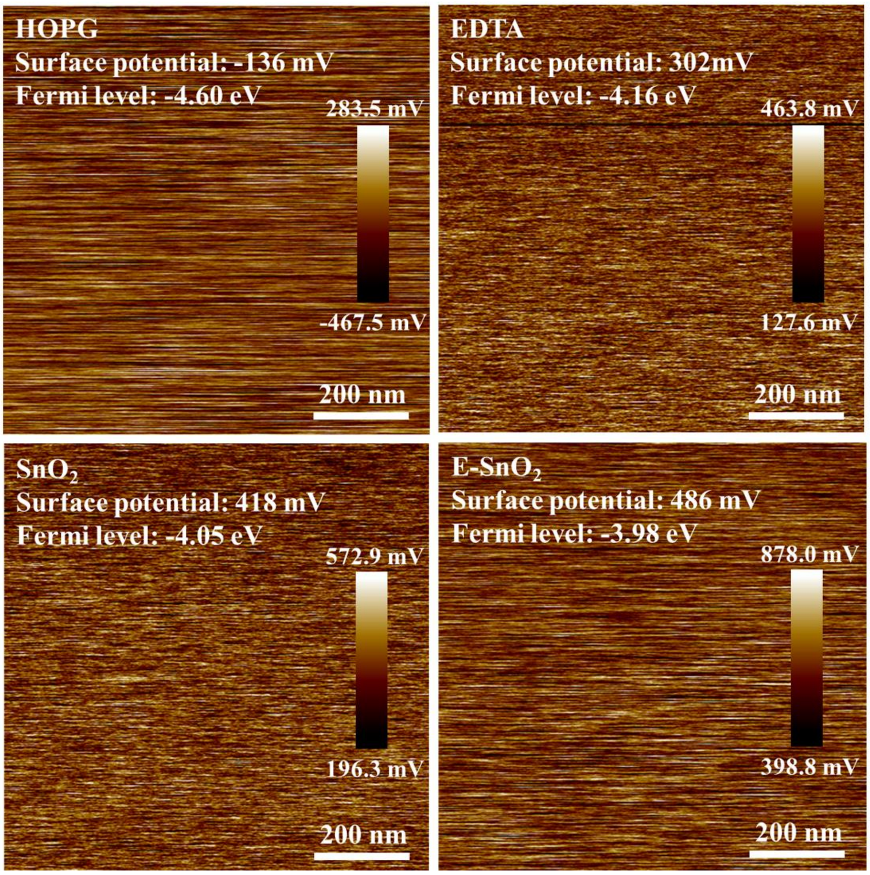

Supplementary Figure 4 | Surface potential images of different films. The surface potential images are crude data without any processing. Their surface potential are measured with KPFM by probing the difference surface potential (SP) between Pt/Ir-coated tip and the samples.[1] The Fermi level of highly ordered pyrolytic graphite (HOPG) is -4.6 eV.[2]

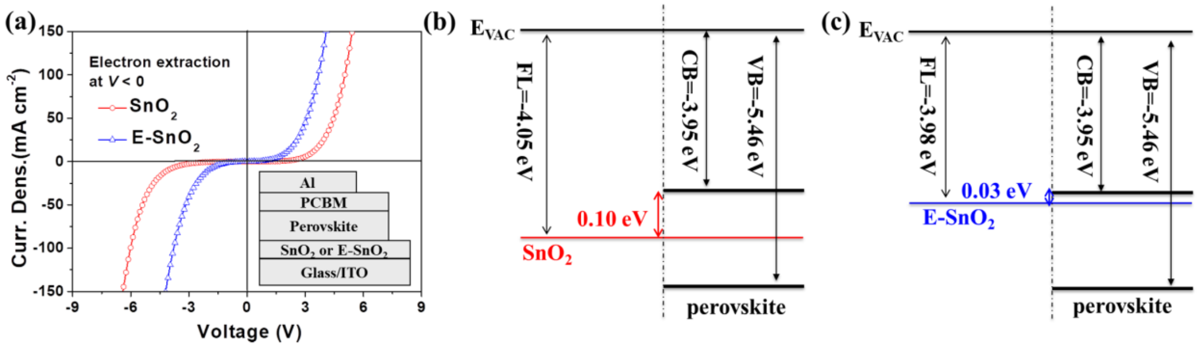

Supplementary Figure 5 | The electron injection capability of SnO_2 and E-SnO_2 ETLs. (a) *J-V* curves of the devices under the dark with SnO_2 and E-SnO_2 ETLs. The inset displays the devices structure in the test. Energy level alignment of perovskite deposited on **(b)** SnO_2 and **(c)** E-SnO_2 substrates. The Fermi level of SnO_2 and E-SnO_2 are obtained by KPFM, and energy levels of $FA_{0.95}Cs_{0.05}PbI_3$ are obtained from the previous report.[3]

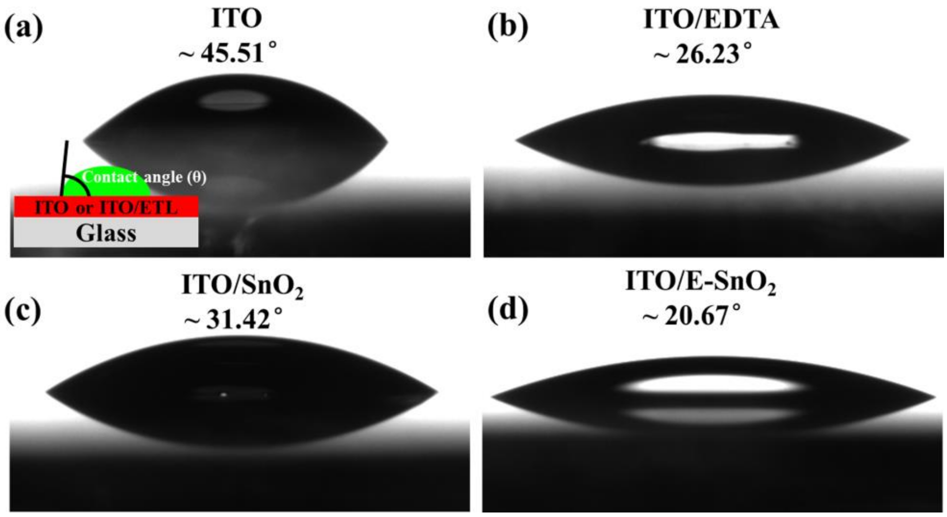

Supplementary Figure 6 | The hydrophilic-hydrophobic property of various substrates. Inset is a definition diagram of the contact angle. The water contact angles of (a) ITO, (b) ITO/EDTA, ITO/SnO_2 and (d) ITO/E-SnO_2.

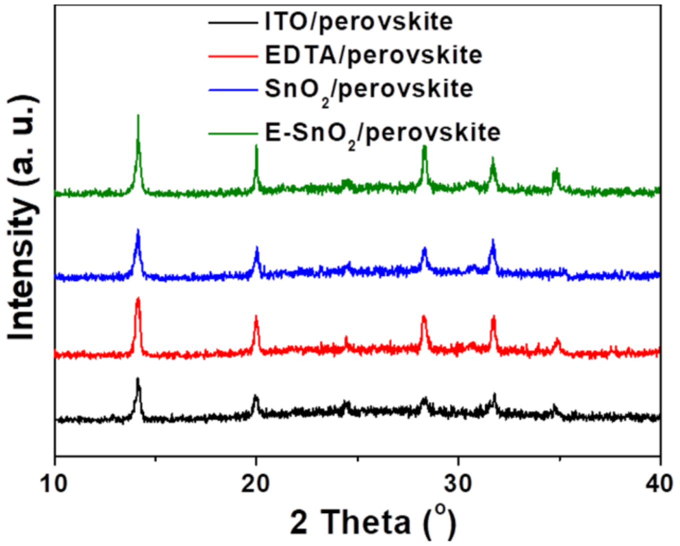

Supplementary Figure 7 | The crystalline of perovskite deposited on different substrates. XRD patterns of perovskite deposited on ITO, EDTA, SnO_2 and E-SnO_2.

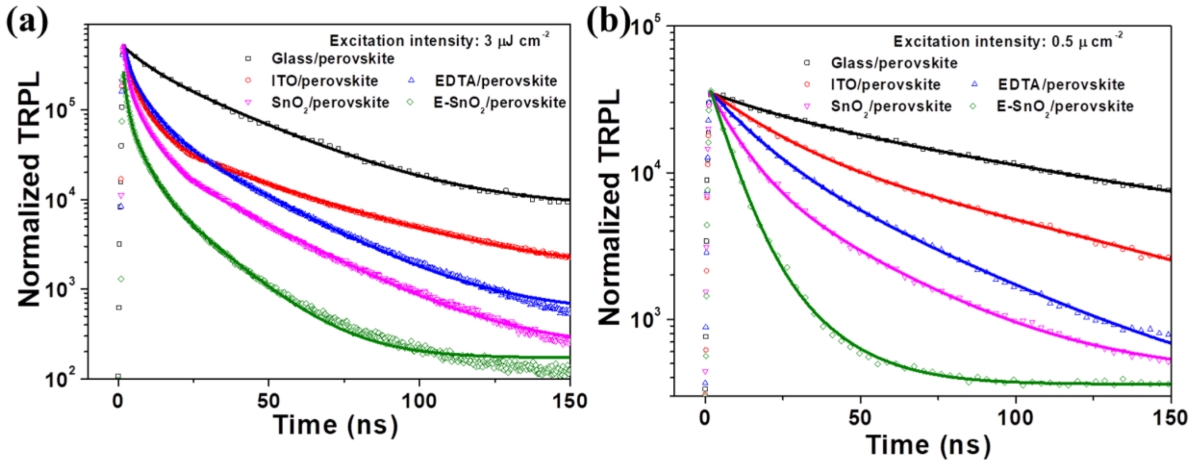

Supplementary Figure 8 | Carrier transport dynamic under different excitation intensity. TRPL spectra of perovskite films deposited on different substrates using excitation intensity of (a) 3 μJ cm^{-2} and (b) 0.5 μJ cm^{-2}.

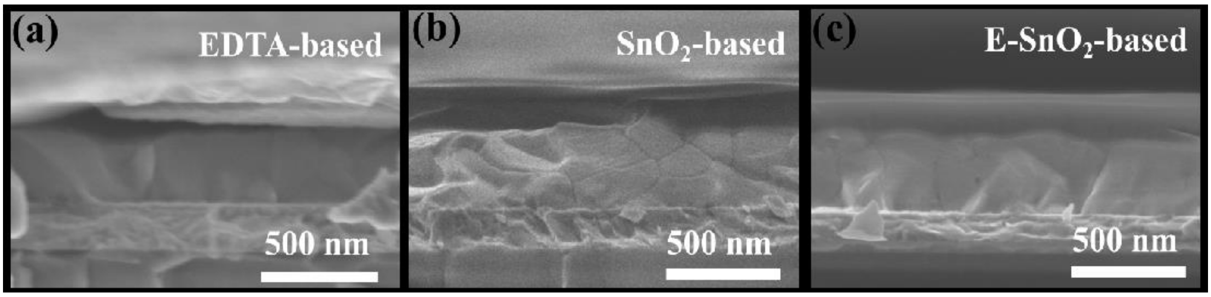

Supplementary Figure 9 | The cross-sectional SEM images of complete planar-type PSCs. (a) EDTA-based **(b)** SnO_2-based and **(c)** E-SnO_2-based perovskite devices measured by SEM technology.

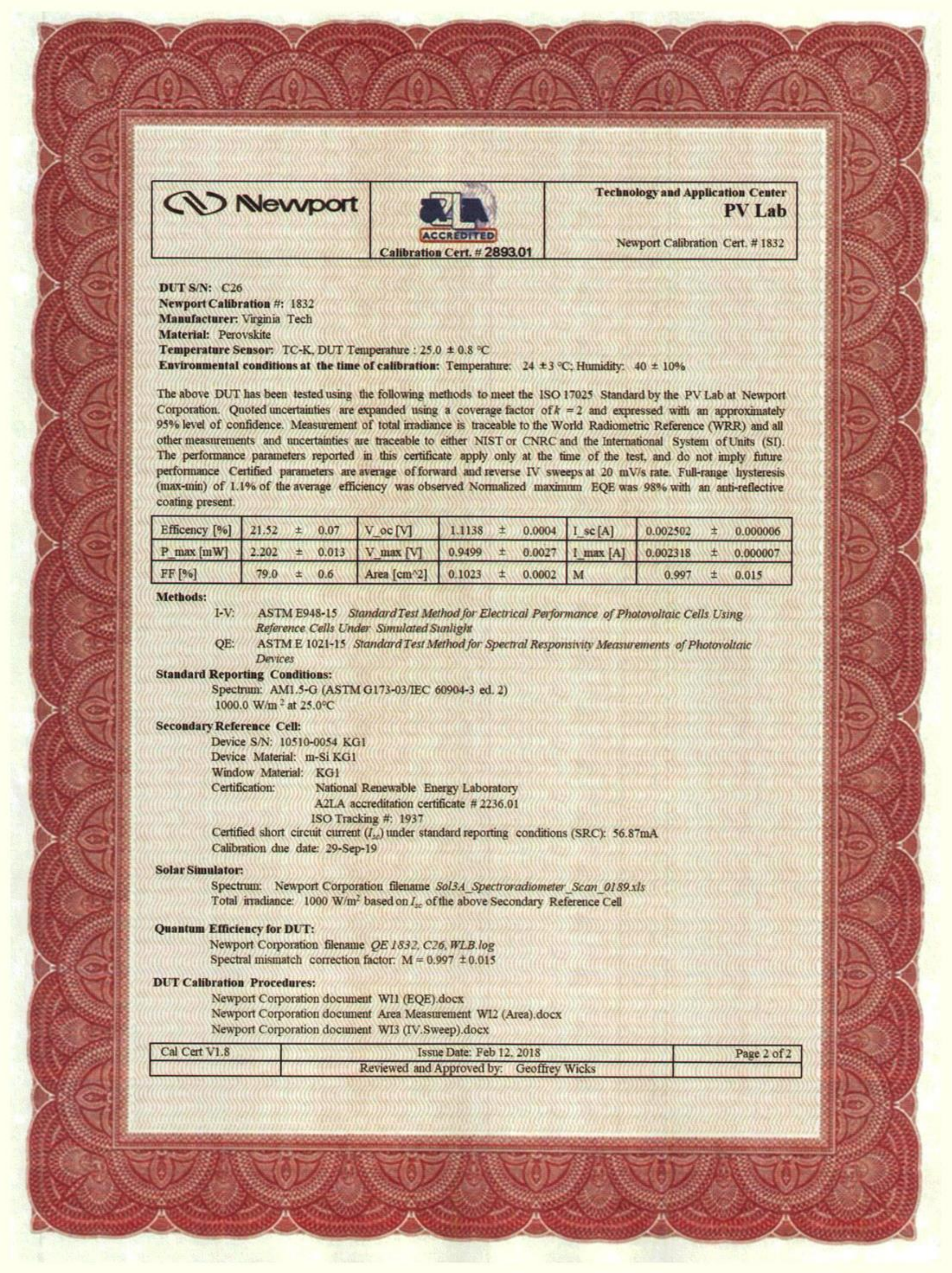

Newport

ACCREDITED

Calibration Cert. # 2893.01

Technology and Application Center

PV Lab

Newport Calibration Cert. # 1832

DUT S/N: C26
Newport Calibration #: 1832
Manufacturer: Virginia Tech
Material: Perovskite
Temperature Sensor: TC-K, DUT Temperature : 25.0 ± 0.8 °C
Environmental conditions at the time of calibration: Temperature: 24 ±3 °C; Humidity: 40 ± 10%

The above DUT has been tested using the following methods to meet the ISO 17025 Standard by the PV Lab at Newport Corporation. Quoted uncertainties are expanded using a coverage factor of $k = 2$ and expressed with an approximately 95% level of confidence. Measurement of total irradiance is traceable to the World Radiometric Reference (WRR) and all other measurements and uncertainties are traceable to either NIST or CNRC and the International System of Units (SI). The performance parameters reported in this certificate apply only at the time of the test, and do not imply future performance. Certified parameters are average of forward and reverse IV sweeps at 20 mV/s rate. Full-range hysteresis (max-min) of 1.1% of the average efficiency was observed. Normalized maximum EQE was 98% with an anti-reflective coating present.

Efficency [%]	21.52 ± 0.07	V_oc [V]	1.1138 ± 0.0004	I_sc [A]	0.002502 ± 0.000006
P_max [mW]	2.202 ± 0.013	V_max [V]	0.9499 ± 0.0027	I_max [A]	0.002318 ± 0.000007
FF [%]	79.0 ± 0.6	Area [cm^2]	0.1023 ± 0.0002	M	0.997 ± 0.015

Methods:
I-V: ASTM E948-15 *Standard Test Method for Electrical Performance of Photovoltaic Cells Using Reference Cells Under Simulated Sunlight*
QE: ASTM E 1021-15 *Standard Test Method for Spectral Responsivity Measurements of Photovoltaic Devices*

Standard Reporting Conditions:
Spectrum: AM1.5-G (ASTM G173-03/IEC 60904-3 ed. 2)
1000.0 W/m^2 at 25.0°C

Secondary Reference Cell:
Device S/N: 10510-0054 KG1
Device Material: m-Si KG1
Window Material: KG1
Certification: National Renewable Energy Laboratory
A2LA accreditation certificate # 2236.01
ISO Tracking #: 1937
Certified short circuit current (I_{sc}) under standard reporting conditions (SRC): 56.87mA
Calibration due date: 29-Sep-19

Solar Simulator:
Spectrum: Newport Corporation filename *Sol3A_Spectroradiometer_Scan_0189.xls*
Total irradiance: 1000 W/m^2 based on I_{sc} of the above Secondary Reference Cell

Quantum Efficiency for DUT:
Newport Corporation filename *QE 1832, C26, WLB.log*
Spectral mismatch correction factor: M = 0.997 ± 0.015

DUT Calibration Procedures:
Newport Corporation document WI1 (EQE).docx
Newport Corporation document Area Measurement WI2 (Area).docx
Newport Corporation document WI3 (IV.Sweep).docx

Cal Cert V1.8	Issue Date: Feb 12, 2018	Page 2 of 2
	Reviewed and Approved by: Geoffrey Wicks	

Supplementary Figure 10 | Certificated results by Newport. The certificated efficiency is 21.52% with J_{sc} = 24.46 mA cm^{-2}, V_{oc} = 1.1138 V and FF = 79.0%, and the mask area is 0.1023 cm^2.

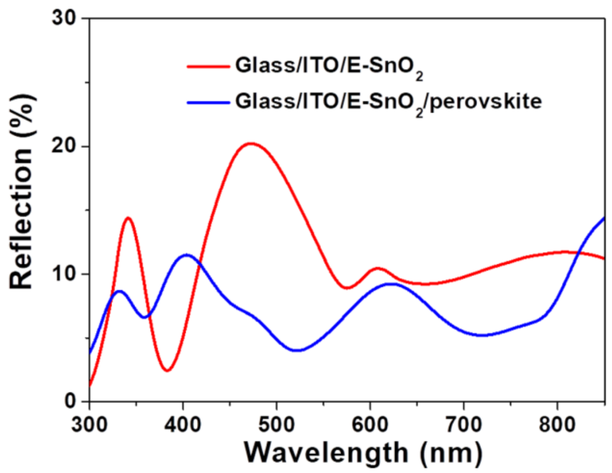

Supplementary Figure 11 | Reflection spectra of the glass/E-SnO_2 and glass/ITO/E-SnO_2/perovskite samples. We characterized the reflection of ITO/E-SnO_2 and ITO/E-SnO_2/perovskite samples, as shown in Supplementary Fig. 11. It can be seen that the average reflection value of ITO/E-SnO_2 is 12.21% in the wavelength range of 400-800 nm. However, when the perovskite absorber layer is deposited onto the ITO/E-SnO_2 substrate, the average reflection is significantly reduced to 6.88%. The reduced optical loss of the ITO/E-SnO_2/perovskite is often seen for multilayer coatings for the antireflection effect due to the difference refractive index between E-SnO_2 (~2.3) and perovskite (~2.9).[5,6] The smaller reflection leads to higher IPCE of the perovskite solar cells based on E-SnO_2 ETLs in the wavelengths from 400 nm to 800 nm.

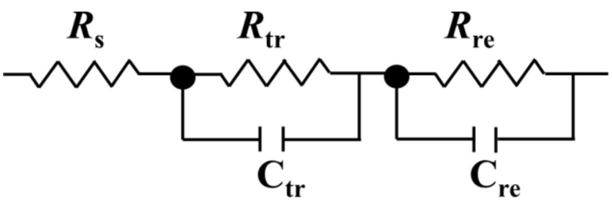

Supplementary Figure 12 | The equivalent circuit model for EIS. The equivalent circuit composed of the series resistance (R_s), the R_{tr} and the R_{rec}.

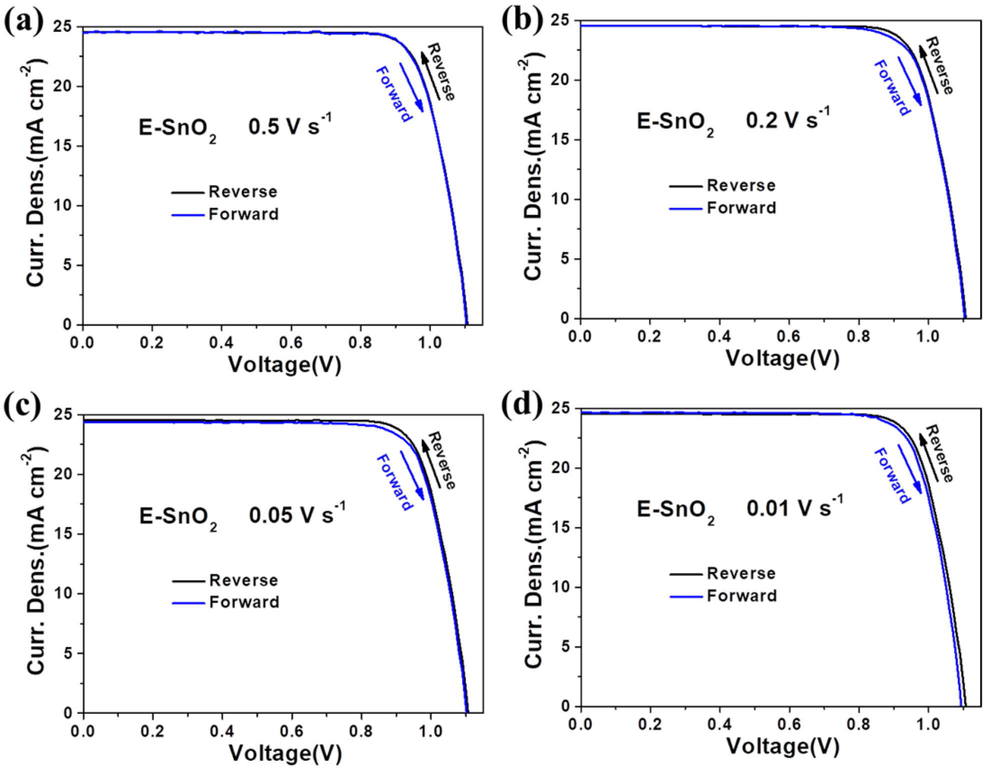

Supplementary Figure 13 | The *J*-*V* curves of planar-type PSCs based on E-SnO_2 at different scan rate. The *J*-*V* curves of device at scan rate of **(a)** 0.5 V s^{-1}, and **(b)** 0.2 V s^{-1}, (c) 0.05 V s^{-1} and 0.01 V s^{-1}.

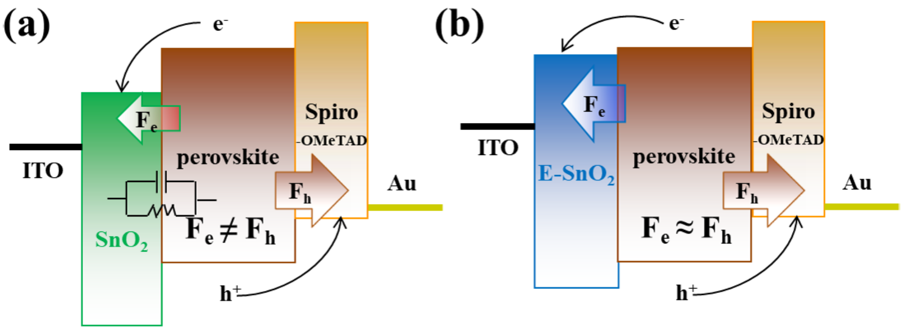

Supplementary Figure 14 | Charge transport mechanism. (a) Planar-type PSCs with SnO_2 and **(b)** E-SnO_2 ETLs.

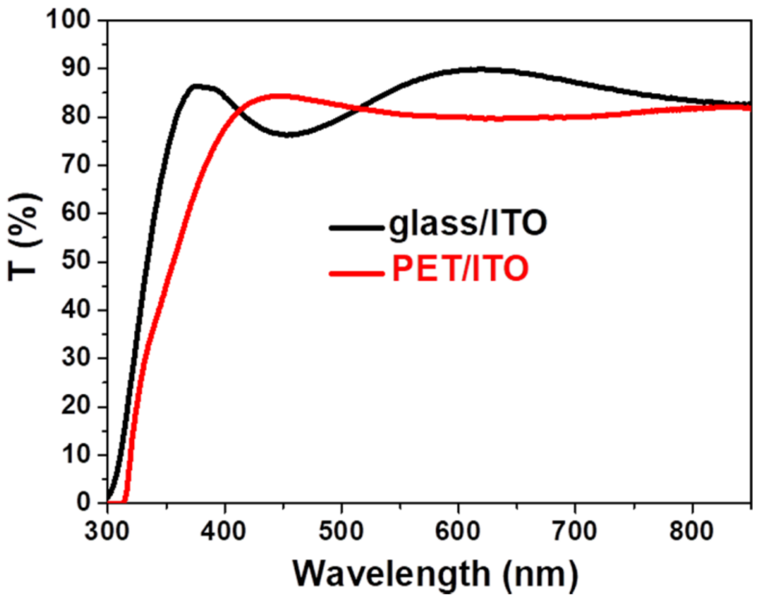

Supplementary Figure 15 | The optical transmittance spectra of glass/ITO and PET/ITO samples. It is clear that the PET/ITO has lower optical transmittance in vision region compared to glass/ITO sample, which leads to less photos generated in perovskite absorber, resulting in low J_{sc} in devices.

Supplementary Table 1 | Parameters of the TRPL spectra of perovskite films deposited on different substrates under various excitation intensity.

Excitation intensity	Sample	τ_{ave} (ns)	τ_1 (ns)	% of τ_1	τ_2 (ns)	% of τ_2
3 μJ cm^{-2}	Glass/perovskite	71.07	76.27	72.35	20.56	27.65
	ITO/perovskite	35.73	36.37	68.24	1.46	31.76
	ITO/EDTA/perovskite	23.04	23.74	65.29	1.44	34.71
	ITO/SnO_2/perovskite	22.52	23.26	57.82	1.09	42.18
	ITO/E-SnO_2/perovskite	13.15	14.16	45.32	0.97	54.68
0.5 μJ cm^{-2}	Glass/perovskite	147.63	149.33	91.72	22.38	8.28
	ITO/perovskite	58.50	65.36	63.33	20.27	36.67
	ITO/EDTA/perovskite	37.71	42.73	60.25	13.35	39.75
	ITO/SnO_2/perovskite	28.57	33.33	52.16	8.71	47.84
	ITO/E-SnO_2/perovskite	13.55	16.40	49.17	6.02	50.83

Supplementary Table 2 | The parameters of PSCs based on EDTA.

Devices	J_{sc} (mA cm^{-2})	V_{oc} (V)	FF	PCE (%)
1	21.25	1.09	0.671	15.56
2	22.32	1.03	0.456	10.50
3	22.33	1.03	0.488	11.17
4	22.94	0.98	0.458	10.33
5	23.66	1.06	0.545	13.71
6	21.90	1.08	0.678	16.11
7	22.10	1.08	0.687	16.42
8	22.14	1.02	0.661	14.86
9	21.36	1.04	0.658	14.62
10	19.76	1.04	0.708	14.55
11	18.61	1.04	0.747	14.46
12	21.38	1.04	0.672	14.94
13	21.21	1.04	0.702	15.48
14	19.89	1.06	0.682	14.38
15	21.32	1.06	0.688	15.55
16	21.28	1.04	0.733	16.22
17	19.44	1.08	0.689	14.47
18	22.29	1.06	0.680	16.07
19	21.97	1.04	0.692	15.81
20	21.63	1.04	0.557	12.53
21	19.13	1.04	0.677	13.47
22	22.12	1.06	0.652	15.29
23	21.03	1.08	0.663	15.06
24	20.35	1.04	0.673	14.24
25	21.32	1.06	0.686	15.50
26	22.38	1.04	0.632	14.71
27	22.41	1.06	0.616	14.63
28	20.32	1.08	0.686	15.05
29	22.89	1.06	0.663	16.09
30	22.13	1.08	0.676	16.16
Average	21.43 ± 1.19	1.05 ± 0.02	0.649 ± 0.074	14.60 ± 1.60

Supplementary Table 3 | The parameters of PSCs based on SnO_2.

Devices	J_{sc} (mA cm^{-2})	V_{oc} (V)	FF	PCE (%)
1	22.81	1.10	0.725	18.19
2	22.74	1.09	0.722	17.92
3	22.60	1.10	0.712	17.62
4	22.95	1.09	0.712	17.90
5	22.92	1.09	0.711	17.79
6	22.16	1.08	0.687	16.46
7	22.25	1.03	0.762	17.51
8	22.28	1.10	0.693	16.94
9	22.59	1.10	0.705	17.54
10	22.94	1.10	0.755	18.93
11	22.70	1.10	0.734	18.34
12	22.61	1.10	0.739	18.35
13	22.99	1.10	0.739	18.63
14	22.65	1.10	0.743	18.52
15	22.90	1.10	0.737	18.60
16	22.80	1.10	0.740	18.55
17	22.16	1.03	0.749	17.08
18	22.98	1.10	0.738	18.66
19	22.73	1.10	0.733	18.33
20	22.86	1.10	0.742	18.62
21	22.87	1.10	0.734	18.47
22	22.94	1.03	0.792	18.78
23	22.84	1.04	0.756	18.01
24	22.71	1.10	0.713	17.82
25	23.40	1.04	0.744	18.03
26	22.30	1.03	0.755	17.41
27	22.88	1.10	0.735	18.50
28	22.07	1.03	0.758	17.31
29	22.33	1.04	0.755	17.47
30	23.20	1.11	0.735	18.87
Average	22.70 ± 0.32	1.08 ± 0.03	0.735 ± 0.022	18.04 ± 0.63

Supplementary Table 4 | The parameters of PSCs based on E-SnO_2.

Devices	J_{sc} (mA cm^{-2})	V_{oc} (V)	FF	PCE (%)
1	24.62	1.11	0.744	20.40
2	24.67	1.13	0.756	20.99
3	24.68	1.12	0.744	20.51
4	24.46	1.12	0.749	20.57
5	25.11	1.11	0.734	20.38
6	24.57	1.11	0.792	21.60
7	23.23	1.12	0.755	19.64
8	23.42	1.12	0.749	19.67
9	24.00	1.12	0.739	19.83
10	24.12	1.11	0.741	19.91
11	24.20	1.11	0.754	20.16
12	24.24	1.10	0.753	20.17
13	24.64	1.10	0.749	20.29
14	25.25	1.09	0.753	20.74
15	25.16	1.09	0.753	20.68
16	25.24	1.09	0.750	20.65
17	24.16	1.08	0.737	19.32
18	25.76	1.10	0.746	21.06
19	25.75	1.10	0.748	21.13
20	25.62	1.12	0.747	21.39
21	24.40	1.12	0.765	20.85
22	25.38	1.11	0.749	21.16
23	23.31	1.11	0.752	19.40
24	23.87	1.10	0.739	19.48
25	25.30	1.11	0.738	20.70
26	25.88	1.10	0.756	21.52
27	24.07	1.11	0.765	20.51
28	24.03	1.11	0.765	20.49
29	23.64	1.11	0.748	19.61
30	23.64	1.11	0.744	19.54
Average	24.55 ± 0.76	1.11 ± 0.01	0.750 ± 0.011	20.41 ± 0.55

Supplementary Table 5 | EIS parameters for the PSCs based on EDTA, SnO_2 and E-SnO_2 ETLs.

PSCs with different ETLs	R_s (Ω)	R_{tr} (Ω)	C_{tr} (F)	R_{rec} (Ω)	C_{rec} (F)
EDTA	61.7	54.6	1.2×10^{-8}	259.9	1.9×10^{-8}
SnO_2	30.1	27.4	1.1×10^{-8}	300.1	1.8×10^{-8}
E-SnO_2	20.3	14.8	2.6×10^{-8}	443.3	1.5×10^{-8}

Supplementary Table 6 | The stability parameters of PSCs based on SnO_2 and E-SnO_2 ETLs without any encapsulation stored at ambient condition, where the relative humidity is about 35%.

ETL	parameters	0 h	96 h	192 h	360 h	552 h	1056 h	1440 h	2160 h	2880 h
SnO_2	J_{sc} (mA cm^{-2})	22.70	22.61	21.96	21.76	21.52	21.32	20.12	19.79	18.81
	V_{oc} (V)	1.10	1.09	1.10	1.10	1.08	1.07	1.08	1.06	1.06
	FF	0.734	0.721	0.719	0.716	0.708	0.710	0.695	0.682	0.678
	PCE	18.34	17.77	17.37	17.14	16.46	16.20	15.10	14.31	13.52
E-SnO_2	J_{sc} (mA cm^{-2})	24.67	24.61	24.47	24.46	24.34	24.55	24.18	24.01	23.93
	V_{oc} (V)	1.13	1.13	1.12	1.13	1.11	1.10	1.12	1.10	1.09
	FF	0.756	0.753	0.761	0.749	0.757	0.755	0.741	0.733	0.738
	PCE	20.99	20.94	20.86	20.70	20.46	20.39	20.07	19.36	19.25

Supplementary Table 7 | The stability parameters of PSCs based on SnO_2 and E-SnO_2 ETLs without any encapsulation tested under continuous one sun condition (100 mW cm^{-2}) in ambient conditions.

ETL	parameters	0 h	5 h	24 h	48 h	72 h	120 h
SnO_2	J_{sc} (mA cm^{-2})	22.61	22.13	21.93	21.32	20.86	19.37
	V_{oc} (V)	1.10	1.09	1.09	1.07	1.06	1.06
	FF	0.739	0.730	0.685	0.665	0.637	0.550
	PCE	18.35	17.62	16.37	15.18	14.09	11.30
E-SnO_2	J_{sc} (mA cm^{-2})	24.40	24.21	24.36	23.82	23.56	23.03
	V_{oc} (V)	1.12	1.12	1.11	1.09	1.09	1.08
	FF	0.765	0.760	0.749	0.756	0.746	0.718
	PCE	20.85	20.62	20.25	19.63	19.16	17.85

Supplementary Table 8 | The parameters of flexible PSCs using E-SnO_2 ETLs.

Devices	J_{sc} (mA cm^{-2})	V_{oc} (V)	FF	PCE (%)
1	23.10	1.10	0.717	18.13
2	23.42	1.09	0.717	18.28
3	23.46	1.10	0.708	18.24
4	22.76	1.08	0.689	17.00
5	22.60	1.07	0.674	16.30
6	22.76	1.07	0.676	16.50
7	22.74	1.09	0.722	17.92
8	22.60	1.10	0.712	17.62
9	22.95	1.09	0.712	17.90
10	22.92	1.09	0.711	17.79
11	22.16	1.08	0.687	16.46
12	21.90	1.08	0.678	16.11
13	22.71	1.10	0.713	17.82
14	22.28	1.10	0.693	16.94
15	21.25	1.09	0.671	15.56
16	22.59	1.10	0.705	17.54
17	22.49	1.10	0.655	16.18
18	22.52	1.12	0.713	18.02
19	22.76	1.11	0.689	17.42
20	22.49	1.11	0.685	17.08
21	22.66	1.11	0.681	17.15
22	23.06	1.11	0.705	18.05
23	22.75	1.09	0.726	17.99
24	22.40	1.09	0.660	16.16
25	22.79	1.08	0.695	17.09
26	22.84	1.04	0.756	18.01
27	22.25	1.03	0.762	17.51
28	23.40	1.04	0.744	18.03
29	22.30	1.03	0.755	17.41
30	22.16	1.03	0.749	17.08
Average	22.64 ± 0.46	1.09 ± 0.03	0.699 ± 0.028	17.26 ± 0.75

Supplementary Note 1.

The Fermi level of samples can be calculated as follows: FL = 4.6 + e(SP_{HOPG} - SP_{sample}), where FL is Fermi level of samples, e is the elementary charge of the electron, SP_{HOPG} and SP_{sample} are surface potential of HOPG and sample, respectively. The 2 × 2 μm^2 scan area is performed on both HOPG and the samples, and the SP of the HOPG, EDTA, SnO_2 and E-SnO_2 are -136, 302, 418 and 486 mV, respectively. The Fermi level of the EDTA, SnO_2 and E-SnO_2 are therefore -4.16, -4.05 and -3.98 eV, respectively.

Supplementary Note 2.

In order to examine electron transport capability of the E-SnO_2 film, the glass/ITO/ETL/perovskite/PCBM/Al devices were fabricated.[4] When a voltage is applied to the top ITO electrode, electrons are injected to ETL from the perovskite. The *J-V* curve of the E-SnO_2-based device exhibits lower response voltage than that of the SnO_2-based device (Supplementary Fig. 5a), indicating that the electron injection from the perovskite to the E-SnO_2 is easier than to the SnO_2, due to the higher electron mobility of E-SnO_2. In addition, Supplementary Fig. b and 5c illustrate the energy level alignment for the devices based on the SnO_2 and E-SnO_2 ETLs. It is clear that the Fermi level of the E-SnO_2 shows a better match to the conduction band of the perovskite than that obtained for the SnO_2. This provides enhancement in the observed V_{oc} (Fig. 4a and Table 1).

Supplementary References

1. Melitz, W. *et al.* Scanning tunneling spectroscopy and Kelvin probe force microscopy investigation of Fermi energy level pinning mechanism on InAs and InGaAs clean surfaces. *J. Appl. Phys.* **108**, 023711 (2010).

2. Zhou, L., Yang, D., Yu, W., Zhang, J. & Li, C. An efficient polymer solar cell using graphene

oxide interface assembled via layer-by-layer deposition. *Org. Electron.* **23**, 110-115 (2015).

3. Ma, F. *et al.* Stable α/δ phase junction of formamidinium lead iodide perovskites for enhanced near-infrared emission. *Chem. Sci.* **8**, 800-805 (2017).

4. Zhou, Y. *et al.* A universal method to produce low-work function electrodes for organic electronics. *Science* **336**, 327-332 (2012).

5. Baco, S. et al. Study on optical properties of tin oxide thin film at different annealing temperature. *J. Sci. Techno.* **4**, 61-72 (2012).

6. Xie, Z. et al. Refractive index and extinction coefficient of $NH_2CH{=}NH_2PbI_3$ perovskite photovoltaic material. *J. Phys.: Condens. Matter* **29**, 245702 (2017).
